# 1265. Penicillin Allergy Delabeling: A Quality Improvement Project

**DOI:** 10.1093/ofid/ofad500.1105

**Published:** 2023-11-27

**Authors:** Ibrahim Shah, Sana Sheraz, Marriam Ali

**Affiliations:** Indiana University School of Medicine, Fishers, Indiana; Indiana University School of Medicine, Fishers, Indiana; Indiana University School of Medicine, Fishers, Indiana

## Abstract

**Background:**

15% of all hospitalized patients report a penicillin allergy, and greater than 90% of them are not truly allergic. Penicillin allergy labels are associated with worse clinical outcomes, increasing in-hospital mortality, and increasing hospital stays. Historically, penicillin allergies were delabeled by either allergists or infectious disease specialists. Pharmacy-led delabeling programs are emerging but are limited due to pharmacists not having provider status in all states. Also, after successful delabeling, a significant number of patients were relabeled for penicillin allergies on subsequent hospital visits.

**Methods:**

We conducted an internal medicine resident-driven quality improvement project in a 350 bed community hospital. Fifty-six patients with a penicillin allergy label in the electronic medical record (EMR) were randomly selected from the inpatient service. If the patient had a non-allergic reaction or had prior exposure to penicillin without a reaction, they were directly delabeled. The remainder underwent risk stratification using the PENFAST scoring tool. Very low-risk and low-risk patients underwent an oral challenge after obtaining written consent. Following this, a hospital-wide pharmacist-triggered physician-driven delabelling protocol was established.

**Results:**

We assessed 56 patients between 04/2022 and 09/2022. Thirty-eight patients were delabeled: 18 by direct delabeling and 20 via oral penicillin challenge. One patient who underwent the oral challenge had an adverse reaction (nausea and vomiting). Seven months later, we conducted a secondary survey to assess the durability of our delabeling. Thirteen percent of patients (n=5) had the penicillin allergy label re-added to their chart on subsequent hospital admissions.

**Conclusion:**

Delabeling patients on the inpatient service is effective, carries little risk, and requires minimal resources. Additionally, it does not require specialist services. However, the durability of delabeling needs to be reinforced with a robust hospital delabeling protocol, continuous patient education, and EMR alerts to prevent allergy labels from being added back on subsequent visits.

**Disclosures:**

**All Authors**: No reported disclosures

